# SARS-CoV-2 Prevalence and Variant Surveillance among Cats in Pittsburgh, Pennsylvania, USA

**DOI:** 10.3390/v15071493

**Published:** 2023-06-30

**Authors:** Santhamani Ramasamy, Abhinay Gontu, Sabarinath Neerukonda, Diana Ruggiero, Becky Morrow, Sheweta Gupta, Saranya Amirthalingam, John M. Hardham, Joshua T. Lizer, Michele Yon, Ruth H. Nissly, Padmaja Jakka, Shubhada K. Chothe, Lindsey C. LaBella, Deepanker Tewari, Meera Surendran Nair, Suresh V. Kuchipudi

**Affiliations:** 1 Department of Veterinary and Biomedical Sciences, Pennsylvania State University, University Park, PA 16802, USA; sqr5895@psu.edu (S.R.);; 2 United States Department of Health and Human Services, Silver Spring, MD 20993, USA; 3 S.R. Scientific LLC, 5854 Ellsworth Ave., Pittsburgh, PA 15232, USA; 4 Frankie’s Friends, 740 5th Ave, New Kensington, PA 15068, USA; 5 Zoetis, Kalamazoo, MI 49007, USA; john.m.hardham@zoetis.com (J.M.H.);; 6 Animal Diagnostic Laboratory, Department of Veterinary and Biomedical Sciences, Pennsylvania State University, University Park, PA 16802, USA; 7 Pennsylvania Department of Agriculture, Pennsylvania Veterinary Laboratory, Harrisburg, PA 17110, USA; 8 Huck Institute of Life Sciences, Pennsylvania State University, University Park, PA 16802, USA

**Keywords:** SARS-CoV-2, cats, antibody, lateral flow assay, pseudovirus neutralization, antigen cartography, surveillance

## Abstract

Severe acute respiratory syndrome coronavirus-2 (SARS-CoV-2) infects many mammals, and SARS-CoV-2 circulation in nonhuman animals may increase the risk of novel variant emergence. Cats are highly susceptible to SARS-CoV-2 infection, and there were cases of virus transmission between cats and humans. The objective of this study was to assess the prevalence of SARS-CoV-2 variant infection of cats in an urban setting. We investigated the prevalence of SARS-CoV-2 variant infections in domestic and community cats in the city of Pittsburgh (*n* = 272). While no cats tested positive for SARS-CoV-2 viral RNA, 35 cats (12.86%) tested SARS-CoV-2-antibody-positive. Further, we compared a cat-specific experimental lateral flow assay (eLFA) and species-agnostic surrogate virus neutralization assay (sVNT) for SARS-CoV-2 antibody detection in cats (*n* = 71). The eLFA demonstrated 100% specificity compared to sVNT. The eLFA also showed 100% sensitivity for sera with >90% inhibition and 63.63% sensitivity for sera with 40–89% inhibition in sVNT. Using a variant-specific pseudovirus neutralization assay (pVNT) and antigen cartography, we found the presence of antibodies to pre-Omicron and Omicron SARS-CoV-2 variants. Hence, this approach proves valuable in identifying cat exposure to different SARS-CoV-2 variants. Our results highlight the continued exposure of cats to SARS-CoV-2 and warrant coordinated surveillance efforts.

## 1. Introduction

The severe acute respiratory syndrome coronavirus-2 (SARS-CoV-2) is the causative agent of the coronavirus disease-2019 (COVID-19) pandemic. The ongoing COVID-19 pandemic has raised concerns about the potential for virus transmission between humans and animals. The attachment and entry of SARS-CoV-2 into host cells predominantly depend on the angiotensin-converting enzyme 2 (ACE2) receptor [1]. Since the ACE2 receptor is conserved among various animal species, SARS-CoV-2 has a remarkable potential to infect a broad range of hosts [2,3]. Computational methods have predicted the likelihood of viral infection across diverse species [3]. Consequently, multiple instances of SARS-CoV-2 spillover infection from humans to animals have been detected across multiple animal species, including felines, canines, white tailed deer, hamsters, minks, and ferrets [4,5,6,7,8,9,10,11,12,13]. Cats, in particular, have been identified as highly susceptible to SARS-CoV-2 infection and involved in cases of virus transmission to humans [14,15]. Since its emergence in Wuhan, China, in late 2019, SARS-CoV-2 has undergone evolutionary changes, resulting in the emergence of various variants such as Alpha, Beta, Gamma, Delta, and Omicron.

The circulation of SARS-CoV-2 in animals has the potential to contribute to the divergent evolution of the virus and the emergence of novel variants. Notably, persistent infection in mink has been associated with the accumulation of mutations in the spike protein [16]. These spike mutations have the potential to confer immune evasion, vaccine failure, and altered host range [16]. Additionally, the persistence of Alpha, Delta, and Gamma variants of concern (VoCs) in white-tailed deer, resulting in the emergence of deer-adaptive mutations, was also documented [17]. Such host-adaptive mutations in SARS-CoV-2 could give rise to new variants that pose an increased risk of transmission and severe illness in humans. The close association between humans and companion animals, including dogs and cats, provides a potential pathway for human-to-animal and animal-to-human transmission. Cats, in particular, are highly susceptible to SARS-CoV-2 infection. Experimental infection of juvenile cats with the SARS-CoV-2 ancestral B.1 lineage has revealed subclinical infections despite virus replication in the respiratory tract, nasal shedding, and transmission to in-contact cats [13]. This suggests that SARS-CoV-2 infection in cats may go unnoticed or undiagnosed, although it could contribute to the spread of the virus within households. Instances of natural human-to-cat transmission of SARS-CoV-2 have been reported [13,18], and there have also been documented cases of cat-to-human transmission [14]. Given the high population densities in urban areas and transmission as a function of exposure and susceptibility, it is reasonable to believe that the probability of human-to-cat transmission would be greater in such settings. Therefore, conducting a surveillance study on cats, with a particular focus on an urban city and employing concentrated geographical sampling, is necessary to assess the prevalence of SARS-CoV-2 in this population.

SARS-CoV-2 infection in animals can be confirmed by detecting viral RNA, viral antigens, or specific antibodies. However, when considering natural infections in animals, selecting appropriate detection methods becomes crucial. Experimental studies have demonstrated that the optimal window for detecting the SARS-CoV-2 antigen or nucleic acid following infection ranges from 2 to 6 or 9 days postinfection [13]. Conversely, antibodies generated in response to SARS-CoV-2 infection can persist in cats for up to 10 months [19]. Given the uncertainty surrounding the exact timing of exposure in natural infections, the utilization of antibody detection tools becomes particularly relevant for SARS-CoV-2 surveillance. These tools enable the identification of past infections, extending beyond the period of active viral shedding. Consequently, antibody detection serves as a valuable approach to assess the prevalence and history of SARS-CoV-2 infections in animal populations [20,21].

The objective of this study was to evaluate the risk of SARS-CoV-2 spillover to cats and their exposure to the virus within the community in an urban setting. To achieve this, we investigated the prevalence of SARS-CoV-2 variant infections in both domestic and community cats (*Felis catus*) in the city of Pittsburgh, located in Allegheny County, western Pennsylvania.

The study period spanned from 4 December 2021 to 3 March 2022. To detect the presence of SARS-CoV-2, we employed reverse transcription quantitative polymerase chain reaction (RT-qPCR) to identify viral RNA. Additionally, we utilized the surrogate virus neutralization test (sVNT) (Genscript, c-pass) for serological surveillance of SARS-CoV-2 in cat sera (*n* = 272) [22]. Furthermore, we assessed the neutralizing activities of sVNT-positive sera against various SARS-CoV-2 lineages, including the ancestral B.1 lineage, Alpha, Beta, Gamma, Delta, and Omicron variants using pseudovirus neutralization assay (pVNT). We also evaluated the diagnostic potential of a feline-specific, unlicensed, experimental-use-only lateral flow antibody detection assay (eLFA) developed at Zoetis. Our study found serological evidence for the exposure of cats to both ancient SARS-CoV-2 variants and the more recent Omicron variant.

## 2. Materials and Methods

### 2.1. Cats and Samples

For our research, we selected Pittsburgh, a city in western Pennsylvania, USA, as a representative urban setting for the study. Samples collected from 272 apparently healthy cats (*Felis catus*) that are either pets or community cats brought to Frankie’s Friends, a nonprofit veterinary clinic and medical rescue for cats in Pittsburgh (Allegheny County), Pennsylvania, for sterilization and submitted to Penn State animal diagnostic laboratory were used in this study. Pet cats are family- or individually owned cats that primarily live indoors, with their owners providing a controlled and protected environment. On the other hand, community cats are unowned, free-ranging cats that live outdoors, often relying on their instincts for survival. Due to their outdoor lifestyle, community cats are more likely to be at a higher risk of exposure to the SARS-CoV-2 virus. In our study, we intentionally included a combination of pet and community cats in the sample population. This approach allowed us to capture animals from different risk levels and better represent the overall cat population. By including both types of cats, we aimed to provide a more comprehensive understanding of SARS-CoV-2 exposure in feline populations, considering the varying lifestyles and potential sources of exposure. The sampling group comprised 57% of tom and 43% of queen cats that belonged to mixed breeds between domestic short-haired and domestic long-haired cats. The estimated ages of cats ranged from 4 months to 6 years. There was no known history of close contact with human patients with respiratory illness.

The samples were collected from 4 December 2021, to 3 March 2022. Cats infected with SARS-CoV-2 may not show any symptoms, which means the apparently healthy cats could have either an ongoing infection or have been exposed to the virus in the past. Oropharyngeal and nasal swabs were collected from each cat in Universal Transport Medium (UTM), and blood was collected for serum separation. Sterile cotton-tipped applicators were used to collect nasal and deep pharyngeal swabs. The oropharyngeal and nasal swabs were used for RNA extraction and RT-qPCR to test for the presence of SARS-CoV-2 RNA, and serum samples were analyzed for SARS-CoV-2-specific antibodies. The swabs were stored in a designated −20 °C freezer, and during RNA extraction, they were handled in a biosafety cabinet (BSL2) following the protocols at the Penn State Animal Diagnostic laboratory approved by the institutional biosafety committee (IBC #48340; Approval Date: 12 October 2022) with the required personnel protective equipment (PPEs) including gloves, disposable coverall, N-95 mask, and face shield.

### 2.2. Quantitative Real Time PCR

RNA was extracted from 400 µL of swab samples using a KingFisher Flex machine (ThermoFisher with the MagMAX Viral/Pathogen extraction kit (Cat no. A42352, ThermoFisher Scientific, Waltham, MA, USA) following the manufacturer’s instructions. OPTI Medical SARS-CoV-2 RT-qPCR kit (IDEXX Laboratories, Inc., Westbrook, ME, USA), targeting the N gene, was used to test the presence of SARS-CoV-2 viral RNA [23,24]. The primers RT-qPCR assays were performed using ABI 7500 Fast instrument (Applied Biosystems, California, USA). The assay was reported to have a limit of detection of 0.9 copies/µL, and the samples with a cycle threshold of 40 or less are considered positive for SARS-CoV-2 RNA. The internal control RNase P was used to confirm that the samples were not contaminated with human tissue or fluids during harvesting or processing.

### 2.3. Surrogate Virus Neutralization Test (sVNT)

Cat sera were tested via sVNT, which detects the binding of SARS-CoV-2-specific antibodies to SARS-CoV-2 RBD (Wuhan) antigens and measures the inhibition of SARS-CoV-2 RBD and human–ACE2 interaction [25]. sVNT is a species agnostic assay that could be used to detect SARS-CoV-2 neutralizing antibodies in serum samples of any species [22,26]. Briefly, diluted serum samples (1:10) were incubated with a horseradish peroxidase conjugated receptor binding domain of SARS-CoV-2 for 30 min at 37 °C. Then the serum–RBD mixture was transferred to ACE2-coated 96-well plates, and the unbound RBDs were allowed to bind to the ACE2. The RBD–ACE2 interactions were measured by using a trimethyl benzidine substrate. The inhibition of RBD binding to ACE2 is proportional to the amount of SARS-CoV-2-specific antibodies in the serum samples. The serum samples with percent inhibition above 30% were declared positive [25,27]. Although we have not tested the prepandemic cat serum samples in this study to determine the threshold for sVNT, the previous research has shown that the 30% inhibition is an optimal cut-off for cat serum [27,28].

### 2.4. Lateral Flow Assay (eLFA)

We used the feline eLFA developed by Zoetis for detecting the SARS-CoV-2-specific antibodies in serum from cats. The eLFA kit contents were equilibrated to room temperature prior to use. Each eLFA result was read visually. Briefly, the sample pad was instilled with a drop of undiluted serum followed by two drops of chase buffer, Zoetis (COVID-19 antibody assay chase buffer for feline). The reagents were allowed to flow horizontally on the work bench for 10 min. The results were considered positive if both test and control lines were visible, negative if only control line was visible, or invalid if the control line was not visible.

### 2.5. Diagnostic Performance of eLFA

We tested the cat serum samples (*n* = 71) in both sVNT and eLFA to determine the diagnostic capability of eLFA. The sensitivity, specificity, accuracy, and predictive values with 95% confidence interval were calculated using diagnostic test evaluation calculator of MedCalc statistical software available online [29].

### 2.6. Production of SARS-CoV-2 Spike Pseudoviruses

SARS-CoV-2 spike pseudoviruses were produced using the third-generation lentiviral packaging plasmids as described elsewhere [30]. The transfer plasmid encoding luciferase and ZsGreen (BEI, Cat no: NR-52516), helper plasmid encoding Gag/pol (BEI, Cat no: NR-52517), and plasmid encoding spike proteins of the SARS-CoV-2 ancestral strain and variants of concern (B.1 lineage, Alpha, Beta, Gamma, Delta, and Omicron BA.1) were transfected in HEK 293T cells using FuGene6 transfection reagent (Promega, Madison, WI, USA. Cat. No. E2691). The HEK 293T cells were propagated using Dulbecco’s Modified Eagle Medium (DMEM) with 10% fetal bovine serum (FBS). The pseudovirus containing supernatants were collected after 48 h of transfection and filtered through 0.45 µ membrane filters, and aliquots were stored at −80 °C until further use. The infectivity of pseudoviruses was tested in HEK 293T cells expressing human ACE2 and TMPRSS2 (BEI, Cat no: NR-55293). Briefly, 1.3 × 10^4^ cells were infected with 10-fold serial dilutions of the pseudoviruses in a 96-well clear bottom white plate (ThermoScientific, Cat. No. 165306). After 48–72 h, the bright-glo luciferase reagent (Promega, Cat. No. E2620) was added to the wells (100 µL per well), the plates were read using BioTek Cytation 5 Cell Imaging Multimode Reader (Agilent, Santa Clara, CA, USA, Cell Imaging Multimode Readers), and relative luminescence units (RLUs) were documented.

### 2.7. Pseudovirus Neutralization Assay (pVNT)

Cat serum samples that were positive in sVNT were tested for the neutralizing activity against SARS-CoV-2 spike pseudoviruses containing spike from the B.1 lineage, or Alpha, Beta, Gamma, Delta, or Omicron variants. pVNTs were performed using HEK 293T cells overexpressing ACE2 and TMPRSS2 (BEI, Cat no: NR-55293), as described earlier [30]. Three-fold serial dilutions of serum samples were incubated with 100 µL of pseudoviruses (equivalent of 10^4^ RLU) at 37 °C for one hour, 5% CO_2_. The serum–antibody mixture was inoculated onto the monolayer of 293T ACE2/TMPRSS2 cells. The pseudovirus infectivity was determined at 48 h postinfection by quantifying luciferase expression. To determine the neutralization titer, we used a normalization process by calculating the percentage of neutralization relative to a virus-only control. This was accomplished using the following formula: 100 − (RLU of sample/RLU of virus control) * 100. The 50% neutralization titer (NT50) for each serum sample was determined by conducting duplicate runs against each pseudovirus. We employed a nonlinear regression curve fitting approach using GraphPad Prism Software version 9.0.0 (San Diego, CA, USA) to model the curves and obtain accurate results.

### 2.8. Antigen Cartography

We utilized NT_50_ measurements of serum samples against SARS-CoV-2 B.1 lineage, and Alpha, Beta, Gamma, Delta, and Omicron VoCs in pVNT to construct antigen cartography [31], as described in previous studies [32]. Antigen cartography software was employed to generate 2D antigenic maps and calculate distances between SARS-CoV-2 VoCs and serum samples [32]. A higher NT_50_ value of serum in relation to a VoC signifies a greater level of antigenic relatedness, resulting in a lower distance. To visualize the antigenic units’ distances between SARS-CoV-2 and serum samples, we utilized GraphPad software for plotting. We performed a two-tailed unpaired Student’s t-test with Welch’s correction for statistical analysis. A *p*-value below 0.05 indicated a significant mean distance between the SARS-CoV-2 VoCs (Wuhan, Alpha, Beta, Gamma, Delta, Omicron) and the serum samples.

## 3. Results

### 3.1. Surveillance of Cats for SARS-CoV-2

**Figure 1:**
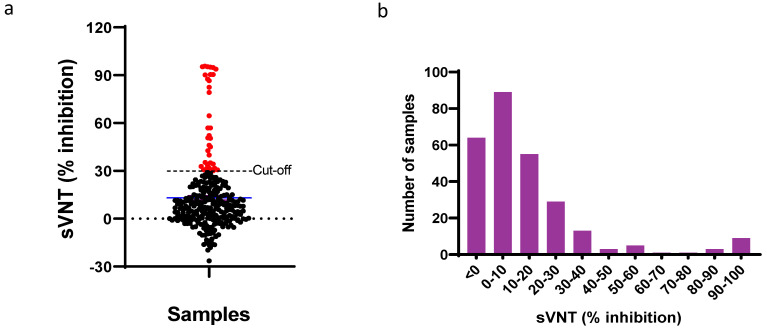
Detection of SARS-CoV-2-specific antibodies by sVNT. (**a**) The distribution of negative (*n* = 237) and positive serum samples (*n* = 35) in surrogate virus neutralization assay (sVNT) with positive–negative cut-off (30%). (**b**) Percentage inhibitions of cat serum samples (*n* = 272) determined by sVNT. Based on the cut-off of 30% inhibition, 237 samples are negative, and 35 are positive for SARS-CoV-2 antibodies.

We analyzed nasal swabs and serum samples from 272 cats to detect SARS-CoV-2 infection. All the nasal swabs (*n* = 272) were tested negative on SARS-CoV-2 N1 gene RT-qPCR. The samples were also negative for RNase P, which indicates that it is highly unlikely that the samples have been contaminated with fluids or tissues from humans. Surrogate virus neutralization test (sVNT) was performed on serum samples to detect SARS-CoV-2-specific neutralizing antibodies. Serum samples with a neutralization titer of 30% or more are considered positive for current or previous SARS-CoV-2 exposure [27]. Out of 272 cats, 35 were positive (neutralization titer >30%), and 237 were negative (neutralization titer <30%) for SARS-CoV-2-specific neutralizing antibodies. Based on sVNT, the cats in this study showed a SARS-CoV-2 seroprevalence rate of 12.86%. The neutralization titer of sVNT-positive cat serum ranged from 30.70 to 95.68% (Figure 1, Table S1).

### 3.2. Diagnostic Performance of eLateral Flow Assay Compared to sVNT

**Figure 2:**
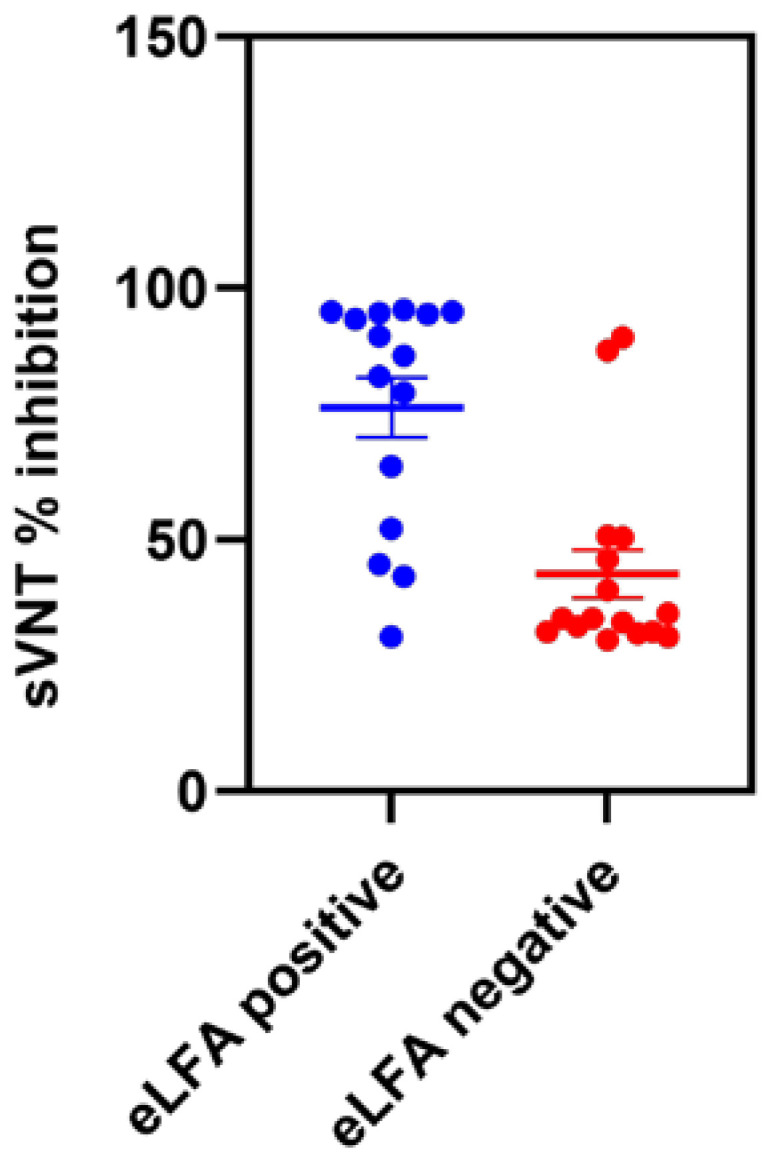
Detection of SARS-CoV-2-specific antibodies in sVNT-positive (>30% inhibition) cat serum samples using eLFA. The error bar indicates standard error of mean.

We tested 31 sVNT-positive (the remaining 4 samples did not have sufficient volume to test) (Table S1) and 40 negative serum samples (Table S1) in a feline eLFA. All the sVNT-negative serum were negative in the feline eLFA, whereas out of 31 positive serum samples, 15 were positive and 16 were negative for SARS-CoV-2 antibodies in eLFA. All the positive samples with >90% inhibition in sVNT were positive in the feline eLFA (100% sensitivity). The remaining serum samples with sVNT inhibition between 40% and 89% had a 63.63% sensitivity in eLFA. Of the 12 weakly positive samples with a sVNT titer between 30% and 39%, only one was positive, indicating an 8% sensitivity (Table 1, Figure 2). The sensitivity of weakly positive samples would likely have been improved using a digital optical reader. The eLFA exhibited a sensitivity of 48.39% in comparison to the sVNT, using a cut-off of 30% (Table 2). The compromised sensitivity was attributed to samples with weak positive results, showing an inhibition range of 30–39% (Figure 2).

### 3.3. SARS-CoV-2 Variant-Specific Antibodies

Serum samples that were positive in the sVNT (*n* = 35) were further tested in a SARS-CoV-2-spike-based pVNT. We employed pseudoviruses with SARS-CoV-2 spike proteins from B.1 lineage, and Alpha, Beta, Gamma, Delta, and Omicron BA.1 variants of concern in the pVNT. The pseudovirus neutralization was not observed in sera with the sVNT neutralization titer ranging from 30% to 35% and one of the sera with 50%. All the other sVNT-positive serum samples (*n* = 22) showed the neutralization of at least one of the SARS-CoV-2 spike pseudoviruses, and an extrapolated serum dilution which had 50% neutralization of pseudoviruses was considered as NT_50_.

**Figure 3:**
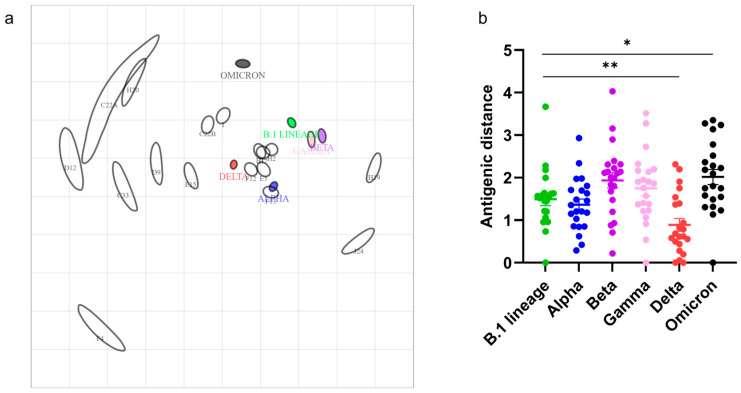
Serum reactivity to SARS-CoV-2 VoCs. (**a**) A 2D antigenic map of SARS-CoV-2 B.1 lineage, and Alpha, Beta, Gamma, Delta, and Omicron variants based on cat serum samples was constructed. SARS-CoV-2 variants are shown as circles, and sera are indicated as squares. The SARS-CoV-2 B.1 lineage, and Alpha, Beta, Gamma, Delta, and Omicron variants are indicated in green, purple, brown, red, blue, and black circles, respectively. Each gray square corresponds to sera from one cat. Both the axes of the map are antigenic distance, and each grid square represents one antigenic unit, which is three-fold serum dilutions (two antigenic units correspond to nine-fold serum dilution and so on) in pseudovirus neutralization assay. The distance between points is a measure of antigenic similarity. The closer points are more similar. (**b**) The antigenic distances between the individual serum and SARS-CoV-2 B.1 lineage, and Alpha, Beta, Gamma, Delta, and Omicron VoCs. The dots indicate antigenic distances of B.1 lineage (green), Alpha (blue), Beta (purple), Gamma (pink), Delta (red), and Omicron (black), respectively. The error bar indicates the standard deviation of the mean distances between SARS-CoV-2 VoC and serum samples. *p*-value of <0.05 indicates the statistical significance. * *p* < 0.05, ** *p* < 0.01.

We used the NT_50_ of serum samples that showed neutralization of at least two variants (*n* = 17) to construct the antigen cartography (Figure 3). The antigen cartography algorithm requires the titers of at least two variants to reliably position the serum in the antigenic map. Each grid square in the antigenic cartography indicates one antigenic unit, which is three-fold serum dilutions in the pVNT. Out of 22 samples analyzed, 16 serum samples had the highest NT_50_-to-Delta VoC. In the antigenic map, these serum samples were positioned near Delta VoC. The mean antigenic distances between serum samples to B.1 lineage, Alpha, Beta, Gamma, Delta, and Omicron were 1.69 ± 0.49, 1.36 ± 0.62, 1.93 ± 0.84, 1.74 ± 0.81, 0.88 ± 0.7, and 2.01 ± 0.83, respectively. Cats positioned close to B.1 lineage, Alpha, Beta, Gamma, and Delta might have been potentially exposed to one of these ancient SARS-CoV-2 variants. In contrast, cats located close to Omicron in the antigenic map might have been exposed to the Omicron variants.

## 4. Discussion

Since the beginning of the COVID-19 pandemic in December 2019, SARS-CoV-2 has been found to infect multiple host species [4,5,18,33]. Although many animal species were reported to be susceptible to SARS-CoV-2, the transmission dynamics, evolution, and genetic adaptation in different hosts are not known. Further, the evolution and emergence of novel SARS-CoV-2 variants could lead to altered host tropism. Therefore, investigating the seroprevalence in animal species would help identify the exposure to SARS-CoV-2 and the risk factors of virus transmission.

Our study aimed to investigate the prevalence of SARS-CoV-2 exposure in two distinct populations of cats: those living in human households (pet cats) and community cats that often come into contact with humans. To ensure the generalizability of our findings, we selected a city in western Pennsylvania as a representative sample of SARS-CoV-2 exposure among cats in other cities throughout the United States. In the USA, 37 million households have cats as pets, the second-most pet population next to dogs [34]. The number of cats in each urban area can differ based on the preferences of pet owners. Based on sVNT, the SARS-CoV-2 seroprevalence in cats (237 negative and 35 positive cats) was 12.86%. Earlier studies reported the seroprevalence of 8% in anti-N antibody detection ELISA and 3% in RBD-ELISA on cat serum samples from Minnesota, USA (*n* = 239) [20]. Schulz and coworkers found a seroprevalence of 4.4% in cat sera samples (*n* = 2160) from Europe during the first wave of COVID-19 [21]. The higher seroprevalence of cat samples observed in our study is consistent with the higher levels of COVID-19 cases reported in humans in Allegany County during the sampling period (Figure S1a). This finding suggests a higher risk of exposure for cats in areas with a higher incidence of COVID-19 cases among the human population. The transmission dynamics of SARS-CoV-2 between humans and cats are known to be influenced by close contact and shared living environments. As humans in the community experience higher infection rates, the likelihood of exposure and transmission to their pet cats increases. The relatively higher seroprevalence (12.86%) in our study could also be due to the samples being collected from both domestic and community cats; community cats potentially have an increased risk of virus transmission from humans and animals.

Sera (*n* = 9), which had a percent inhibition of 30–35% in sVNT, did not show pseudovirus neutralization; it could be because the low level of antibodies was not detectable at the lowest dilution (1:40) we tested in pVNT. Except for these serum samples, all the sera showed neutralization of at least one or more SARS-CoV-2 spike pseudoviruses (B.1 lineage, Alpha, Beta, Gamma, Delta, and Omicron). About 72% of serum samples had the highest titer and lowest antigenic distance to the SARS-CoV-2 Delta variant but had significant cross-neutralization ability to Alpha and B.1 lineage compared to Beta and Gamma variants. These cats might have been exposed to single or multiple spillovers of Delta or any of the ancient variants from humans. These cats could have been infected with SARS-CoV-2 B.1 lineage, or Alpha, Beta, Gamma, or Delta variants. It is known that the cross-protection of these variants to Omicron is lower [35]. One of the cats tested had neutralization titer only to SARS-CoV-2 Omicron variants and positioned close to Omicron in the antigenic map. This indicates that the cat was likely infected with SARS-CoV-2 Omicron. The detection of pre-Omicron and Omicron antibodies in cats is consistent with the ongoing human-to-cat spillover of SARS-CoV-2. The surveillance of SARS-CoV-2 variants in humans in Allegheny County during the study period (Figure S1b) has shown the circulation of these specific variants, and our findings indicate that cats have been exposed to and developed antibodies against these variants. These results suggest that pVNT coupled with antigen cartography could be valuable in surveilling SARS-CoV-2 in animals as it helps identify past infections by different virus variants.

Our study has several limitations. Firstly, the small sample size used in this study may restrict the generalizability of our findings. Another limitation is that our study relied on opportunistically collected diagnostic samples obtained during routine health monitoring of cats. This approach may introduce selection bias and does not represent a truly random sample of the feline population. Consequently, our findings may not accurately reflect the overall prevalence of SARS-CoV-2 in cats. Furthermore, it is worth noting that we did not detect any actively infected cats in our study. This could be attributed to the small sample size, and our samples were primarily obtained from clinically healthy animals. To address these limitations, future research should incorporate well-designed epidemiological studies that encompass larger and more diverse cat populations. Random sampling methods should be employed to obtain a representative sample, allowing for a more accurate estimation of SARS-CoV-2 prevalence in feline populations.

While the clinical significance of infection of cats with the recent SARS-CoV-2 variants remains unclear, the detection of antibodies against Omicron and pre-Omicron variants suggests that human-to-cat spillover of SARS-CoV-2 continues. It is unclear whether cats, like humans exposed to earlier SARS-CoV-2 variants, could be reinfected with recent variants. Our findings highlight the possibility of cats serving as potential reservoirs for the virus and contributing to its spread within the community. Transmission from cats to humans can perpetuate the circulation of variants, even in the presence of mitigation measures against human-to-human transmission. The persistent presence of SARS-CoV-2 in cats and intermittent spillover events could increase the risk of novel variant emergence [36,37].

The feline eLFA developed at Zoetis demonstrated 100% specificity compared to sVNT. The eLFA demonstrated 100% sensitivity with sera showing >90% inhibition and 63.63% sensitivity with sera showing 40–89% inhibition in sVNT. This study suggests the assay may be useful for field application for sero-surveillance and diagnosis based on antibody detection; however, such use would require approval by USDA and/or other applicable agencies. Further investigations are required to understand the diagnostic capability of the feline eLFA for the detection of antibodies elicited by recent variants.

The remarkable ability of SARS-CoV-2 to infect multiple nonhuman animal hosts is a significant aspect of its zoonotic nature. The virus has demonstrated a broad host range, with documented infections in various animal species, including domesticated animals, wildlife, and even some captive zoo animals [38]. When viruses spillover into a new host, adaptive mutations at the interhost level will increase replication and facilitate onward transmission in the new hosts [39]. Therefore, viruses rapidly adapt through random mutagenesis or due to replication and selection in novel host species [40]. Unmonitored circulation of SARS-CoV-2 in animals leads to divergent evolution and emergence of novel variants [36]. For example, a cryptic SARS-CoV-2 lineage was identified on two mink farms in Poland in late 2022 and early 2023 [37]. The study’s authors proposed that the cryptic SARS-CoV-2 lineage may have originated from an unknown or undetected animal reservoir. In addition, older or immunocompromised animals may develop chronic or persistent infections, allowing rapid evolution of SARS-CoV-2 as observed in infected immunocompromised human patients [41,42].

Companion animals, such as cats and dogs, are considered at a greater risk of SARS-CoV-2 infection due to their close proximity to humans and the potential for direct transmission [43]. Hence, there is an urgent need for a One Health approach to coordinate surveillance efforts in humans, animals, and the environment to mitigate the potential risk to human and animal health [44].

In conclusion, our study revealed a prevalence of 12.86% for SARS-CoV-2 antibodies among cats in Pittsburgh, with evidence of exposure of cats to both pre-Omicron and Omicron variants. Our results contribute to understanding SARS-CoV-2 dynamics in the feline population and provide valuable insights into the performance of diagnostic methods.

## Figures and Tables

**Table 1:** Comparison of sVNT and eLFA.

Sample Call	sVNT (% Inhibition)	sVNT; Number of Samples	eLFA		% Accuracy
			Positive	Negative	
Negative	0–29	40	0	40	100%
Positive	30–39	12	1	11	8.30%
	40–49	3	2	1	66%
	50–69	4	2	2	50%
	70–89	4	3	1	75%
	90+	8	8	0	100%

**Table 2:** Diagnostic performance of eLFA.

sVNT Status	Total Samples	Positive	Negative	Sensitivity (95% CI)	Specificity (95% CI)	Positive Predictive Value (95% CI)	Negative Predictive Value (95% CI)	Accuracy (95% CI)
Positive	31	15	16	48.39% (30.15% to 66.94%)	100.00% (91.19% to 100.00%)	100.00% (78.20% to 100.00%)	71.43% (57.79% to 82.70%)	77.46% (66.00% to 86.54%)
Negative	40	0	40					

## Data Availability

The data presented in this study are available on request from the corresponding author.

## Supplementary Materials

The following supporting information can be downloaded at the following: https://www.mdpi.com/article/10.3390/v15071493/s1, Table S1: Detection of SARS-CoV-2 antibodies in cats using sVNT, pVNT, and eLFA. Figure S1: (a) The number of confirmed SARS-CoV-2 cases per day in Allegheny County in humans in Allegheny County at the time of sampling (4 December 2021 to 3 March 2022). (b) Heatmap with the percentage of confirmed SARS-CoV-2 variants in humans (scale represents 0 to 100%). References [45,46] are cited in the supplementary materials.
