# Supplementary material for: SARS-CoV-2 Prevalence and Variant Surveillance among Cats in Pittsburgh, Pennsylvania, USA
Source: Viruses. 2023 Jun 30;15(7):1493. doi: 10.3390/v15071493 (PMC10386599; doi:10.3390/v15071493)
Supplement: Supplementary file 1 [file viruses-15-01493-s001.zip › viruses-2457278-supplementary.pdf]

viruses MDPI

Supplementary Materials:

Table S1. Detection of SARS-CoV-2 antibodies in cats using sVNT, pVNT and eLFA.

S.no	ID	sVNT positive	NT$_{50}$ titer (Pseudovirus neutralization)						pVNT status	eLFA results
			Wuhan	Alpha	Beta	Gamma	Delta	Omicron		
1	E - 5	93.82	2133	6233	1915	1971	3527	1369	Pos	Pos
2	D 12	45.09	-	-	-	-	66.17	36.07	Pos	Pos
3	F 13	95.02	1091	3856	917.5	1263	2024	259.2	Pos	Pos
4	F 7	90.13	740.1	1569	577.4	507.2	1073	628.8	Pos	Neg
5	F 4	52.17	-	43.24	-	-	109.4	-	Pos	Pos
6	D 9	64.62	163.2	141	49.68	37.03	500.1	-	Pos	Pos
7	F 12	90.43	1372	3141	795.1	1368	3888	579.8	Pos	Pos
8	C - 22a	42.67	-	34.75	-	-	118.6	-	Pos	Pos
9	M	46.05	36.56	-	-	-	-	-	Pos	Neg
10	N	32.86	-	-	-	-	34.66	-	Pos	Neg
11	V1	34.35	-	-	-	32.46	-	-	Pos	Neg
12	V2	34.35	-	-	-	-	-	32.53	Pos	Neg
13	C - 22b	94.76	328.7	186.6	168.6	151.9	752.2	378	Pos	Pos
14	U	30.70	-	-	-	-	-	-		Neg
15	Y	31.67	-	-	-	-	-	-		Neg
16	I	95.68	405.30	273.8	177.6	238.6	560.7	510.7	Pos	Pos
17	CC	50.45	-	-	-	-	-	-		Neg
18	C-10	39.89	-	-	-	-	31.86	-	Pos	Neg
19	J	30.63	-	-	-	-	-	-		Pos
20	EE	35.32	-	-	-	-	-	-		Neg
21	H	33.61	-	-	-	-	-	-		Neg
22	C	30.03	-	-	-	-	-	-		Neg
23	J-24	82.38	120.6	165.3	134.9	190.2	103.3	-	Pos	Pos
24	H-19	86.51	393.9	183.0	577.1	219.3	111.2	41.7	Pos	Pos
25	H-7	95.27	1770.0	1805.0	1544.0	2238.0	3804.0	718.7	Pos	Pos
26	H-2	95.37	3953.0	2202.0	1902.0	1947.0	3918.0	623.8	Pos	Pos
27	H-20	50.65	57.5	30.3	39.4	48.1	193.0	-	Pos	Neg
28	E-15	79.15	347.6	150.9	104.5	151.1	597.6	80.0	Pos	Pos
29	C-33	87.54	61.16	-	-	36.17	148.1	54.26	Pos	Neg
30	NN	31.37	-	-	-	-	-	-		Neg
31	O	31.67	-	-	-	-	-	-		Neg

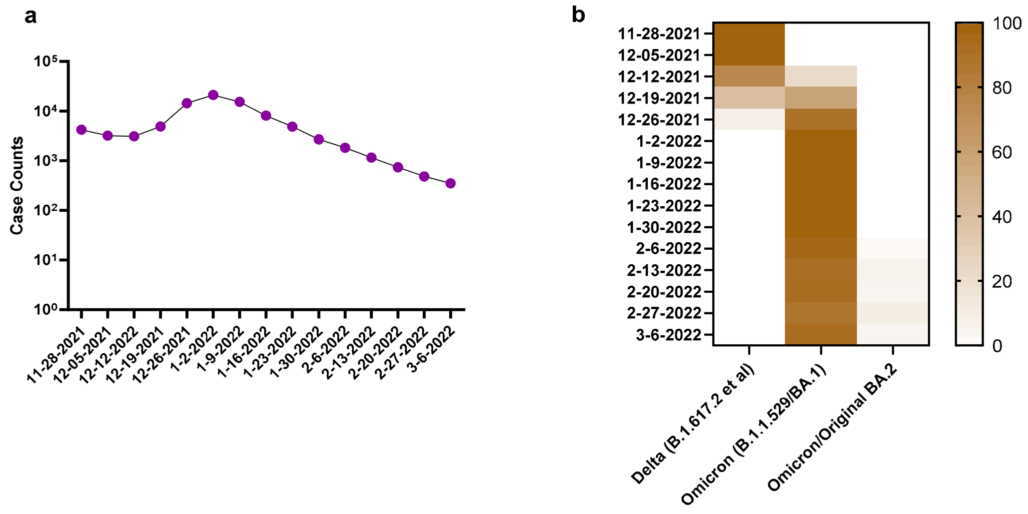

Figure S1. a. The number of confirmed SARS-CoV-2 cases per day in Allegheny County in humans at the time of sampling (December 4, 20221 to March 3, 2022)) **[1]**. **b**. Heatmap with the percentage of confirmed SARS-CoV-2 variants in humans (scale represents 0 to 100%) **[2]**.

References

1. COVID-19 in Allegheny County. https://tableau.alleghenycounty.us/t/PublicSite/views/COVID-19Summary_16222279737570/COVID-19Summary?%3AshowAppBanner=false&%3Adisplay_count=n&%3AshowVizHome=n&%3Aorigin=viz_share_link&%3AisGuestRedirectFromVizportal=y&%3Aembed=y (June 13, 2023),
2. Variants and Genomic Surveillance for SARS-CoV-2 in Allegheny County. https://tableau.alleghenycounty.us/t/PublicSite/views/COVID-19-VariantandGenomicSurveillance_16523720460910/VariantsandGenomicSurveillanceforSARS-CoV-2?%3Aembed=y&%3AshowAppBanner=false&%3Adisplay_count=n&%3AshowVizHome=n&%3Aorigin=viz_share_link (June 13, 2023),
